# Remission of Psychotic Symptoms Following Seizures in a Patient With an Episodic Psychotic Disorder

**DOI:** 10.7759/cureus.104667

**Published:** 2026-03-04

**Authors:** Roli R Gupta, Santosh Ramdurg

**Affiliations:** 1 Psychiatry, Shri. B. M. Patil Medical College, Hospital and Research Centre, Vijayapura, IND

**Keywords:** electroconvulsive therapy, episodic psychotic disorder, psychosis, remission, schizophrenia spectrum disorder, seizures

## Abstract

The relationship between psychosis and seizure activity has long been of clinical interest and historically contributed to the development of convulsive therapies in psychiatry. We report the case of a 36-year-old woman with an episodic psychotic disorder who presented with a relapse characterized by persecutory delusions. She was initiated on antipsychotic treatment, with partial early improvement in symptoms. During the course of hospitalization, her illness was complicated by two episodes of generalized seizures associated with fever and altered consciousness, necessitating intensive care management and ventilatory support. After recovery from this acute medical illness, the patient demonstrated complete remission of psychotic symptoms, which persisted at follow-up. This case highlights a striking temporal association between seizure occurrence and remission of psychosis and underscores the complex and poorly understood relationship between psychosis and seizure-related brain dysfunction. While no causal or therapeutic role of seizures is implied, this observation mirrors similar reports in the literature and emphasizes the importance of continued medical vigilance in the management of acute psychotic episodes.

## Introduction

Psychosis is a clinical syndrome characterized by impaired reality testing and may include delusions, hallucinations, disorganized thought processes, and behavioral disturbances [1]. Psychotic symptoms occur across a range of psychiatric disorders, including schizophrenia spectrum disorders, schizoaffective disorder, brief psychotic disorder, and mood disorders with psychotic features [1]. Some psychotic illnesses demonstrate an episodic course, with periods of exacerbation followed by partial or complete remission, and considerable variability in symptom severity, duration, and treatment response across episodes [1,2]. Examples of such disorders include schizophrenia, schizoaffective disorder, and related psychotic illnesses in which relapses alternate with phases of relative clinical stability [1,2].

Persecutory delusions are fixed false beliefs that one is being harmed, monitored, or conspired against despite evidence to the contrary, whereas auditory hallucinations involve perception-like experiences of hearing voices or sounds in the absence of external stimuli. These symptoms represent common manifestations of psychotic disorders and may fluctuate during the course of illness [1].

Isolated clinical reports have described improvement or remission of psychotic symptoms following spontaneous or treatment-associated seizures [3,4]. However, these observations remain uncommon, and it is unclear whether such outcomes reflect seizure-related neurobiological effects, pharmacological treatment response, systemic illness, or the natural episodic course of psychiatric disorders.

The relationship between psychosis and seizure activity has long been of clinical and theoretical interest. Early observations suggesting interactions between seizure states and psychotic symptoms contributed historically to the development of convulsive therapies, including electroconvulsive therapy (ECT), which remains an established treatment for selected psychiatric conditions [5,6]. Contemporary understanding recognizes that the relationship is complex and likely mediated by multifactorial neurobiological mechanisms rather than a direct causal link [7].

We present the case of a 36-year-old woman with an episodic psychotic disorder whose relapse was complicated by generalized seizures and severe systemic illness, after which sustained remission of psychotic symptoms was observed. This case highlights the complex interplay between psychosis, systemic illness, and seizure-related brain dysfunction and emphasizes the importance of continued medical vigilance during acute psychiatric presentations.

## Case presentation

A 36-year-old woman presented to the psychiatry service with a relapse of psychotic symptoms characterized predominantly by persecutory delusions. Family members reported increasing suspiciousness over several days before presentation, with beliefs that others intended to harm her. At admission, there was no documented history of fever, altered consciousness, or seizure activity. Physical and neurological examinations were unremarkable.

She had experienced a previous psychotic episode approximately one year earlier, during which she reported auditory hallucinations and persecutory beliefs. That episode remitted with pharmacological treatment, and she remained asymptomatic for several months; however, medications were discontinued approximately two months after remission. Detailed records of prior medication dosages were unavailable at the time of current admission. According to information obtained from the patient and family members, developmental history was unremarkable, with no reported childhood behavioral disturbances. There was no documented history of major medical illness, neurological disorders, or previous seizures. No history of substance use, significant trauma, or forensic issues was reported, and family psychiatric history was non-contributory based on available information. She was married, living with family members, and had maintained satisfactory social and occupational functioning before the current episode. Premorbidly, she was described as sociable, responsible, and independent in activities of daily living, with no clearly documented premorbid personality abnormalities.

Mental status examination at presentation revealed suspiciousness, persecutory ideation, and limited insight. Hallucinations were not clearly elicited during the initial assessment. Baseline laboratory investigations, including complete blood count, renal and liver function tests, serum electrolytes, thyroid function tests, and blood glucose levels, were within normal limits. No acute abnormalities were identified on initial clinical evaluation.

She was diagnosed with a relapse of an episodic psychotic disorder and commenced on olanzapine and valproate, with dosages adjusted clinically according to tolerability and response. Over the following two days, overt psychotic symptoms appeared to lessen; however, she became progressively drowsy and less interactive.

On the third day of hospitalization, she developed a generalized tonic-clonic seizure lasting approximately 15 seconds, associated with urinary incontinence and postictal confusion. A second similar episode occurred subsequently, followed by fever and worsening sensorium. Due to a declining level of consciousness and concern for airway protection, she required intubation and transfer to the intensive care unit (ICU). Glasgow Coma Scale (GCS) scores were reduced during the acute phase, although exact values were not consistently documented.

**Figure 1:**
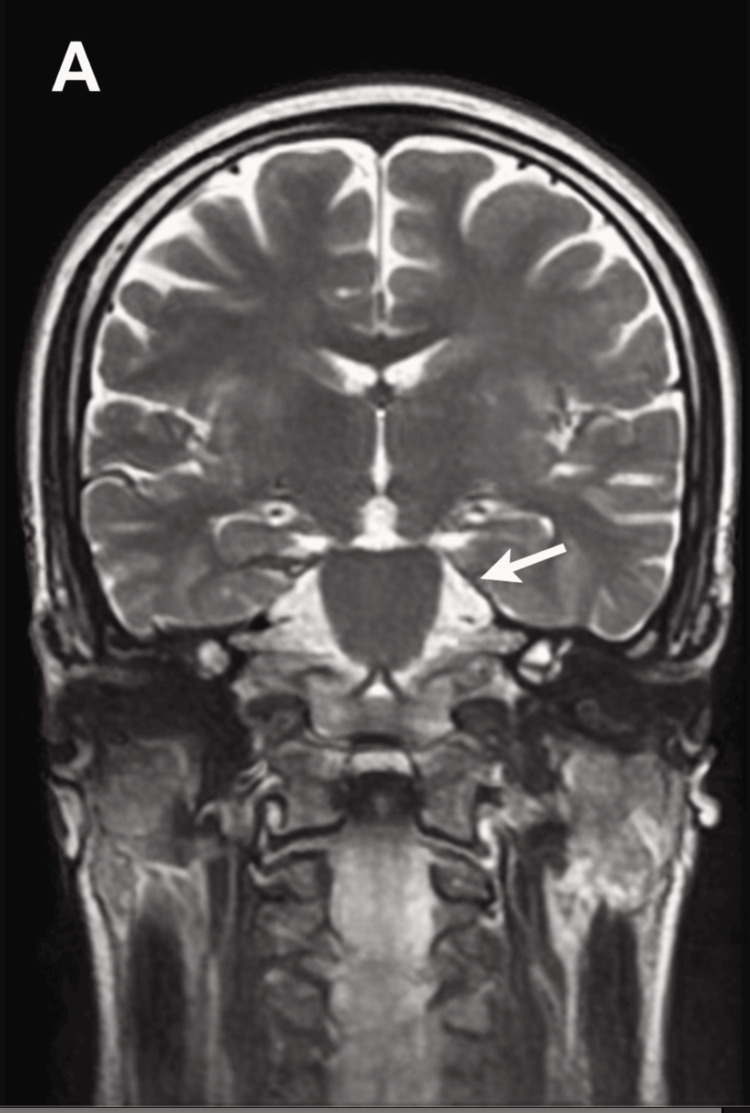
Brain magnetic resonance imaging (MRI). Axial T2-weighted brain MRI demonstrating no acute intracranial abnormalities. The arrow indicates a representative region of normal brain parenchyma.

**Figure 2:**
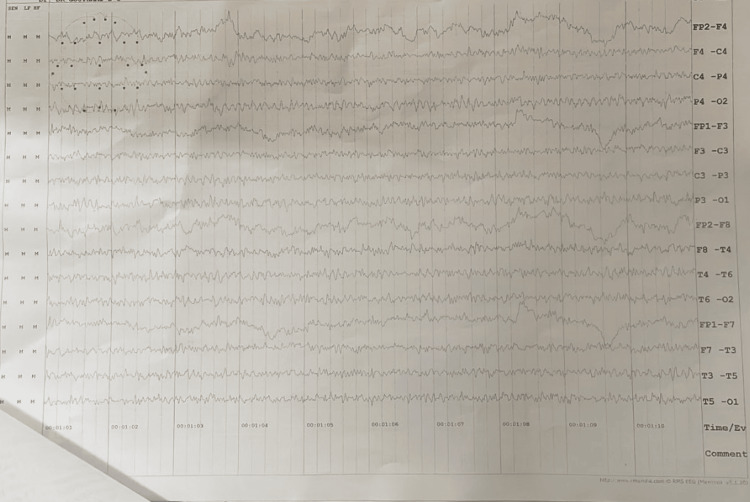
Electroencephalography (EEG) recording with no epileptiform discharges.

Magnetic resonance imaging (MRI) of the brain demonstrated no acute intracranial abnormalities (Figure 1). Electroencephalography (EEG) showed no epileptiform discharges (Figure 2). Laboratory investigations revealed markedly elevated inflammatory and muscle injury markers, including C-reactive protein and creatine phosphokinase-MB, consistent with a significant systemic stress response. No definitive infectious or structural etiology was identified. Cerebrospinal fluid analysis was not performed. Relevant laboratory, endocrine, and neuroimaging investigations are summarized in Table 1.

**Table 1:** Summary of laboratory and neurodiagnostic findings.

Investigation	Result	Reference range/Expected finding	Interpretation
C-reactive protein	90 mg/L	<5 mg/L	Markedly elevated
Creatine phosphokinase-MB	616 U/L	<25 U/L	Markedly elevated
Serum calcium	8.7 mg/dL	8.5–10.5 mg/dL	Normal
Thyroid function tests	Within normal limits	Thyroid-stimulating hormone, T3, T4 within reference range	Normal
Complete blood count	Within normal limits	—	Normal
Serum electrolytes	Within normal limits	—	Normal
Renal function tests	Within normal limits	—	Normal
Liver function tests	Within normal limits	—	Normal
Random blood glucose	Within normal limits	—	Normal
Electroencephalogram	Normal	No epileptiform activity	Normal
Magnetic resonance imaging of the brain	Normal	No structural abnormality	Normal

She received supportive ICU management, including ventilatory support, intravenous fluids, antiepileptic treatment, and close clinical monitoring. Psychiatric medications were adjusted during critical care according to medical stability. Over several days, the fever resolved, and consciousness improved, allowing extubation. No further seizures occurred during hospitalization.

Following recovery, the patient was alert, oriented, and cooperative. Notably, persecutory delusions and other psychotic symptoms were no longer evident. She was discharged after clinical stabilization on maintenance psychiatric treatment with planned outpatient follow-up.

At follow-up several weeks after discharge, she remained free of psychotic symptoms, had no recurrent seizures, and had resumed routine daily activities with satisfactory social functioning.

## Discussion

The most striking feature of this case was the complete remission of psychotic symptoms following recovery from the seizure-associated acute illness. Before hospitalization, the patient had prominent persecutory ideation, whereas after the acute episode and subsequent stabilization, she exhibited no active psychotic features and remained in remission at follow-up.

Psychotic disorders with an episodic course are known to demonstrate variability in symptom severity, duration, and treatment response across episodes, and spontaneous or treatment-associated remission may occur as part of the natural course of illness [1,2]. Therefore, although the temporal association observed in this case is noteworthy, causal inference must be made cautiously.

Similar observations have been reported in the literature. Jawaid et al. described a patient with treatment-resistant schizophrenia whose psychotic symptoms remitted abruptly following a clozapine-induced seizure [3]. Likewise, Park and Lee reported improvement in refractory schizophrenia in the context of seizure activity associated with clozapine treatment and ECT [4]. Although our patient did not have treatment-resistant schizophrenia and was not receiving clozapine, the temporal association between seizure occurrence and remission of psychotic symptoms is comparable to these reports.

Historically, the relationship between psychosis and seizure activity has been of clinical interest and contributed to the conceptual development of convulsive therapies such as ECT [5,6]. Contemporary understanding suggests that seizure activity may influence widespread neural networks, neurotransmitter systems, neuroendocrine pathways, and neuroplastic processes, potentially altering psychiatric symptom expression [7]. However, these mechanisms remain incompletely understood, and direct extrapolation from seizure occurrence to therapeutic benefit is not supported.

A seizure is defined as a transient occurrence of signs and/or symptoms resulting from abnormal, excessive, or synchronous neuronal activity in the brain [8]. Generalized seizures involve bilateral cortical networks and are commonly associated with impaired consciousness and motor manifestations. Importantly, the seizures observed in the present case occurred in the context of acute systemic illness and therefore differ from epilepsy, which requires recurrent unprovoked seizures for diagnosis [9].

An important distinguishing feature of this case is the severity of the associated systemic illness. The patient developed fever, altered sensorium, and required intensive care management with ventilatory support. Laboratory investigations demonstrated markedly elevated inflammatory and muscle injury markers, indicating a significant systemic stress response, while MRI of the brain and EEG were unremarkable. Increasing evidence suggests that systemic inflammation and physiological stress can transiently affect brain function through immune-mediated pathways, metabolic disturbances, and alterations in cortical excitability, potentially influencing psychiatric symptom expression during recovery [10]. This highlights that profound but potentially reversible neurobiological disturbances may occur even in the absence of demonstrable structural or electrophysiological abnormalities.

In the present case, the remission of psychotic symptoms is more plausibly explained by the combined effects of antipsychotic treatment, the natural episodic course of the illness, and global neurobiological changes associated with severe systemic illness and recovery. Nevertheless, the close temporal relationship between seizure episodes and symptom remission remains clinically intriguing and aligns with the small body of published case literature describing similar phenomena [3,4].

It is important to emphasize that this observation should not be interpreted as supporting any therapeutic or beneficial role of seizures in psychotic disorders. Rather, this case adds to the limited literature highlighting the complex and incompletely understood relationship between psychosis, systemic illness, and seizure-related brain dysfunction. Future research should focus on prospective studies examining seizure-associated changes in psychotic symptomatology, neuroinflammatory mechanisms, and network-level brain dynamics to clarify whether these observations reflect direct neurobiological effects or coincidence within the natural course of illness [11].

## Conclusions

This case describes an episodic psychotic disorder with an unusual clinical trajectory in which remission of psychotic symptoms was observed following seizure episodes and recovery from severe acute systemic illness. While the temporal association is clinically striking, no causal or therapeutic role of seizures can be inferred. The observed remission is more plausibly explained by the combined effects of antipsychotic treatment, the natural episodic course of illness, and global neurobiological changes associated with systemic illness and recovery. This case highlights the complex and incompletely understood relationship between psychosis, systemic medical illness, and seizure-related brain dysfunction, and underscores the importance of maintaining medical vigilance when patients presenting with acute psychosis develop changes in neurological or physical status. Further prospective research is needed to clarify the mechanisms underlying such observations and their clinical significance.
